# Hepatic dysfunctions in COVID-19 patients infected by the omicron variant of SARS-CoV-2

**DOI:** 10.3389/fpubh.2022.1049006

**Published:** 2022-11-18

**Authors:** Jianguo Zhang, Daguo Zhao, Jianhui Hu, Xing Huang, Qingqing Gu, Zhimin Tao

**Affiliations:** ^1^Department of Emergency Medicine, The Affiliated Hospital of Jiangsu University, Zhenjiang, China; ^2^Department of Critical Care Medicine, The First Affiliated Hospital of Soochow University, Suzhou, China; ^3^Department of Laboratory Medicine, Zhenjiang Hospital Affiliated to Nanjing University of Chinese Medicine, Zhenjiang Hospital of Traditional Chinese Medicine, Zhenjiang, China; ^4^Center for Evidence-Based and Translational Medicine, Zhongnan Hospital of Wuhan University, Wuhan, China; ^5^Department of Infectious Diseases, The Affiliated Hospital of Kangda College of Nanjing Medical University, The Fourth People's Hospital of Lianyungang, Lianyungang, China; ^6^Jiangsu Province Key Laboratory of Medical Science and Laboratory Medicine, School of Medicine, Jiangsu University, Zhenjiang, China

**Keywords:** SARS-CoV-2, omicron variant, COVID-19, hepatic injury, scoring system

## Abstract

**Background:**

Presently, the omicron variant of severe acute respiratory syndrome coronavirus 2 (SARS-CoV-2) dominates amid the coronavirus disease 2019 (COVID-19) pandemic, but its clinical characteristics with intrinsic severity and organ tropism remain understudied.

**Methods:**

We reported 1,001 mild COVID-19 patients that were infected with the omicron variant of SARS-CoV-2 and hospitalized in China from February to June 2022, including their demographic information, medical/immunization history, clinical symptom, and hematological profile. Patients with one-, two- and three-dose vaccination were compared to assess the vaccine effectiveness. Importantly, liver damage caused by the omicron variant infection was evaluated, in comparison to that caused by the wild-type or the delta variant SARS-CoV-2 infection.

**Results:**

For the reported COVID-19 patients infected by the omicron variant of SARS-CoV-2, their median age was 36.0 [interquartile range (IQR): 26.0-50.0] and 49.7% were female. Hypertension, diabetes, and bronchitis were the leading comorbidities, and asymptomatic patients took up a major portion (61.2%). While most hematological parameters revealed the alleviated pathogenicity, full vaccination or booster shot showed effective protection against clinical severity. Furthermore, liver damages caused by viral infection of the omicron variant were largely attenuated when compared to those by infection of the wild-type or the delta variant SARS-CoV-2.

**Conclusions:**

Our results supported that the viremic effect of the omicron variant tended to be modest, while the liver damage caused by this strain became milder than the previous circulating variants.

## Introduction

The coronavirus disease 2019 (COVID-19) has flamed up across the world for more than two years, and the medical attention has been focused on the pulmonary disorders induced by its responsible pathogen, i.e., severe acute respiratory syndrome coronavirus 2 (SARS-CoV-2) (1, 2). Undoubtedly, COVID-19 is a respiratory illness where most complications are intrapulmonary, typified by viral pneumonia and so triggered acute respiratory distress syndrome (3, 4). However, an array of extrapulmonary dysfunctions has been intensively reported, including those in neural, cardiovascular, and renal systems, revealing that COVID-19 is far more than a lung disease (5–7). Among them, liver impairments in COVID-19 patients are continuously documented, albeit the etiological mechanism remains little known (8).

Since the early outbreak of SARS-CoV-2, the clinical manifestations in a substantial portion of COVID-19 patients have demonstrated the prominent liver injury, mostly determined by abnormal elevations of biomarker enzymes in their sera (9, 10). The types of liver damage are either hepatocellular when represented by heightened levels of alanine aminotransferase (ALT) and aspartate aminotransferase (AST), or cholestatic when characterized by increased concentrations of alkaline phosphatase (ALP), γ-glutamyl transferase (GGT) and bilirubin, or both (mixed) (11). In parallel, although the major histopathologic lesions identified from autopsy studies were located in the respiratory tracts of COVID-19 victims, inflammatory signs in the livers, such as periportal lymphocyte infiltration, sinusoidal microthrombi, and multifocal hepatic necrosis, were commonly observed (12, 13).

Through rapid genetic mutations, SARS-CoV-2 has experienced numerous variants from the ancestral strain, and among them the alpha, beta, gamma, delta, and omicron forms were designated as variants of concern (VOCs), each later one emerging with increased transmissibility, infectivity, and immune escape capacity (14–17). While the disease severity may differ upon the infection of different viral variants, the profiles of organ injuries in COVID-19 patients may concomitantly vary. Moreover, the possibly changing pathological profiles regarding organ tropisms, due to the serial waves of COVID-19 outbreaks induced by the evolving SARS-CoV-2 variants, remained an uncharted area. Thereby, contextualized in the COVID-19 resurges, we here investigated the clinical characteristics of patients infected by the omicron variant of SARS-CoV-2 and accentuated their hepatic dysfunction when compared to those induced by the wild type or the delta variant.

## Methods

### Patient information

The retrospective study included 1,001 COVID-19 patients who were admitted at the Fifth People's Hospital of Suzhou (TFPHS, the Affiliated Infectious Diseases Hospital of Soochow University) and The Fourth People's Hospital of Lianyungang (TFPHL), both in Jiangsu Province, China, from February 9 to June 5, 2022. COVID-19 infections were diagnosed and confirmed as reported (18, 19). Briefly, diagnosis was made based on a combination of epidemiological history, clinical symptom, and laboratory test, where a positive nucleic acid detection of SARS-CoV-2, confirmed by reverse transcription polymerase chain reaction (RT-PCR) testing of patient samples from nasopharyngeal and oropharyngeal swabs, is the primary diagnostic criterion. Exclusion criteria were as follows: patients with malignancy, pregnancy, blood disease, or autoimmune deficiency, and patients having a previous history of liver diseases (including viral hepatitis, liver cirrhosis, and hepatic failure and liver injury caused by medications), gallstones, cholecystitis or encephalopathy, and patients who failed to complete blood examinations. The study was approved by the Research Ethics Commission of TFPHS and TFPHL, respectively. Patient information remained anonymous, and written consents were waived due to a major infectious disease outbreak. All patients were recovered and discharged, and no patients were developing into severe or critically ill conditions.

### Procedure and vaccination

Once COVID-19 patients infected by the omicron variant of SARS-CoV-2 were confirmed, they were isolated and hospitalized for treatment as reported (19, 20). In general, according to patient condition, mild adult COVID-19 patients were treated with antiviral drugs, including nirmatrelvir/ritonavir tablet (Paxlovid), ambavirumab/romisevirumab injection, and intravenous immunoglobulin, etc. For most of admitted COVID-19 patients, two types of inactivated vaccines (Sinovac or Sinopharm) have been administered. Serological tests of patients based on detection of SARS-CoV-2-specific immunoglobulin M (IgM) and immunoglobulin G (IgG) were conducted, using 2019-nCoV Ab test kit (colloidal gold detection manufactured by Innovita Biological Technology Co. Ltd., China, or chemiluminescence immunoassay assays kit manufactured by Shenzhen YHLO Biotech Co., Ltd., China). Scoring systems that are conventionally evaluated for advanced liver diseases, including AST to platelet ratio index (APRI), Child-Pugh, Fibrosis-4 (FIB-4), and Model for End-stage Liver Disease (MELD), have been applied for COVID-19 prognosis upon hospital admission (21–25).

### Statistical analysis

Data were summarized as the median and interquartile range (IQR) values for continuous variables and frequencies for categorical variables. For comparisons between two groups, Mann-Whitney U test was used for continuous variables. Categorical variables were examined by χ^2^ test. All calculated *p-*values were two-sided, and *p*-values <0.05 were considered statistically significant. All statistical analyses were performed using SPSS version16.0 (SPSS Inc., Chicago, IL).

## Results

### Baseline clinical characteristics of COVID-19 patients infected by the omicron variant of SARS-CoV-2

In this study 1,001 COVID-19 patients infected by the omicron variant of SARS-CoV-2 were hospitalized from February to June 2022 in Jiangsu Province, China. Their median age was 36.0 (IQR: 26.0–50.0), and 50.3% were male (Table 1). We then grouped the patients into three; one with none or partial (one-dose) vaccination (164 patients, 16.4%), one with full (two-dose) vaccination (533 patients, 53.2%), and one that had received booster shots (three-dose vaccination) (304 patients, 30.3%). Next, the demographic information, medical history, clinical symptom, and antibody response were analyzed for all patients, together with comparisons of those baseline characteristics between patients none/partially vaccinated and patients fully vaccinated (indicated by *p*^1^ values), and between patients fully vaccinated and patients three doses vaccinated (indicated by *p*^2^ values) (Table 1).

**Table 1:** Demographic information, medical/immunization history, clinical symptom, and antibody production in the COVID-19 patients infected by the omicron variant of SARS-CoV-2.

	**Total (*n* = 1,001)**	***p* ^1^**	**None or partially vaccinated (*n* = 164)**	**Fully vaccinated (*n* = 533)**	**Three doses vaccinated (*n* = 304)**	***p* ^2^**
Age (year)	36.0 (26.0–50.0)	0.002	38.0 (26.3–56.3)	32.0 (18.5–49.0)	40.0 (32.0–50.0)	< 0.0001
Gender, female (%)	498 (49.7)	0.495	79 (48.2)	273 (51.2)	146 (48.0)	0.374
**Comorbidity (%)**						
Hypertension	109 (10.9)	0.011	25 (15.2)	45 (8.4)	39 (12.8)	0.042
Diabetes	41 (4.1)	0.067	10 (6.1)	16 (3.0)	15 (4.9)	0.155
Bronchitis	8 (0.8)	0.020	5 (3.1)	3 (0.6)	0 (0)	0.557
Cardiovascular diseases	8 (0.8)	0.630	2 (1.2)	4 (0.8)	2 (0.7)	1.000
Hypothyroidism	1 (0.1)	0.235	1 (0.6)	0 (0)	0 (0)	1.000
**Symptoms**						
Asymptomatic	613 (61.2)	0.231	104 (63.4)	310 (58.2)	199 (65.5)	0.038
Fever	241 (24.1)	0.991	45 (27.4)	146 (30.8)	50 (16.5)	< 0.001
Cough	197 (19.7)	0.060	23 (14.0)	110 (20.6)	64 (21.1)	0.887
Sore throat	92 (9.2)	0.089	8 (4.9)	48 (9.0)	36 (11.8)	0.189
Expectoration	61 (6.1)	0.088	5 (3.1)	36 (6.8)	20 (6.6)	0.922
Fatigue	53 (5.3)	0.427	12 (7.3)	30 (5.6)	11 (3.6)	0.195
Diarrhea	13 (1.3)	0.226	4 (2.4)	5 (0.9)	4 (1.3)	0.730
Vomiting	9 (0.9)	0.400	3 (1.8)	5 (0.9)	1 (0.3)	0.426
Abdominal pain	3 (0.3)	0.553	1 (0.6)	2 (0.4)	0 (0)	0.537
**Antibody production (%)**						
None	580 (57.9)	< 0.0001	142 (86.6)	358 (67.2)	80 (26.3)	< 0.0001
Only IgG	419 (41.9)	< 0.0001	22 (13.4)	173 (32.5)	224 (73.7)	< 0.0001
Only IgM	7 (0.7)	0.596	0 (0)	5 (0.9)	2 (0.7)	1.000
IgG+IgM	5 (0.5)	1.000	0 (0)	3 (0.6)	2 (0.7)	1.000

Comparisons were performed between patients none/partially vaccinated and patients fully vaccinated (exhibited by *p*^1^ values), between patients fully vaccinated and patients who were three doses vaccinated (exhibited by *p*^2^ values).

Among all patients, hypertension, diabetes, and bronchitis were the leading comorbidities (other minor comorbidities less than 1% were not listed). 81.1% patients had no known pre-existing diseases. In addition to those with typical symptom of fever, cough, sore throat, expectoration, and fatigue, etc., asymptomatic patients occupied more than half of total infections. Regardless of immunization status, 57.9% COVID-19 patients infected by the omicron variant did not develop antibody response while 41.9% producing only IgG, leaving 0.7 and 1.2% patients yielding only IgM and IgG+IgM, respectively.

Compared to patients who had been none/partially vaccinated, patients who had been fully vaccinated or received boost shot owned a much lower ratio of no antibody production and a much higher ratio of producing IgG. Notably, patients who had received the booster shot showed higher occurrence of asymptomatic infection.

### Blood parameters of COVID-19 patients infected by the omicron variant of SARS-CoV-2

A substantial portion of the omicron COVID-19 patients demonstrated abnormal levels of white blood cells (WBCs), neutrophils, lymphocytes, and monocytes, suggesting notable leukocytosis, neutrophilia, lymphocytopenia and monocytosis as cellular signs of SARS-CoV-2 omicron variant infection (Table 2). However, for most omicron COVID-19 patients, the count of red blood cells (RBCs), and the levels of hemoglobin and hematocrit (HCT) remained within the normal range, indicating that anemia was insignificant. This was further supported by the evidence that RBC distribution width (RDW) in most patients remained regular, confirming the minimal damage on erythrocytes in omicron COVID-19 patients. Furthermore, thrombocytopenia was marginal with only 3.8% patients having abnormality in the platelet count, implying the minor effect of the omicron variant infection on platelet. Concurrently, coagulopathy was found in a modest amount of omicron COVID-19 patients, typified by the example that the D-dimer levels in the majority of patients fell in the normal range, still imparting 10.8% patients with abnormally high values. Thus, as we investigated the viremic effect of omicron variant on blood profiles of patients, moderate hematological impairment was observed.

**Table 2:** Baseline characteristics of COVID-19 patients infected by the omicron variant in their hematological profiles.

	**Normal range**	**Total** **(*n* = 1,001)**	***P* ^1^**	**None or partially vaccinated** **(*n* = 164)**	**Fully vaccinated** **(*n* = 533)**	**Three doses vaccinated** **(*n* = 304)**	***p* ^2^**
**Blood cell count**							
WBCs, × 10^9^/L	3.5–9.5	6.08 (4.81–7.55)	0.546	5.79 (4.55–7.36)	5.97 (4.80–7.46)	6.45 (4.97–7.83)	0.020
	>9.5	88 (8.8)	0.179	17 (10.4)	38 (7.1)	33 (10.9)	0.063
Neutrophils, × 10^9^/L	1.8–6.3	4.25 (2.93–5.60)	0.447	3.97 (2.67–5.41)	4.15 (2.82–5.49)	4.58 (3.31–5.98)	0.002
	>6.3	172 (17.2)	0.344	29 (17.7)	78 (14.6)	65 (21.4)	0.013
Lymphocytes, × 10^9^/L	1.1–3.2	1.03 (0.70–1.56)	0.229	0.95 (0.60–1.55)	1.04 (0.71–1.60)	1.04 (0.74–1.48)	0.719
	< 1.1	542 (54.2)	0.500	92 (56.1)	283 (53.1)	167 (54.9)	0.608
Monocytes, × 10^9^/L	0.1–0.6	0.54 (0.40–0.70)	0.013	0.58 (0.43–0.75)	0.53 (0.39–0.68)	0.53 (0.42–0.68)	0.584
	>0.6	391 (39.1)	0.022	77 (47.0)	197 (37.0)	117 (38.5)	0.661
RBCs, × 10^12^/L	3.8–5.1	4.70 (4.38–5.13)	0.092	4.60 (4.29–5.12)	4.71 (4.40–5.09)	4.78 (4.38–5.21)	0.190
	< 3.8	18 (1.8)	0.128	6 (3.7)	9 (1.7)	3 (1.0)	0.552
Hemoglobin, g/L	115–150	139.0 (129.0–153.0)	0.178	135.0 (126.0–151.8)	138.0 (129.0–150.5)	142.5 (132.0–157.0)	0.002
	< 115	57 (5.7)	0.099	14 (8.5)	27 (5.1)	16 (5.3)	0.901
HCT, %	35–50	41.20 (38.29–44.90)	0.287	40.50 (37.32–44.55)	40.90 (38.10–44.48)	41.95 (38.87–46.18)	0.006
	< 35	68 (6.8)	0.079	16 (9.8)	31 (5.8)	21 (6.9)	0.529
MCV, fL	82–100	87.80 (84.60–90.80)	0.756	87.45 (84.23–91.25)	87.40 (84.35–90.30)	88.40 (85.73–91.00)	0.004
	< 82	130 (13.0)	0.529	26 (15.9)	74 (13.9)	30 (9.9)	0.090
MCH, pg	27–34	29.70 (28.60–30.80)	0.865	29.55 (28.35–30.90)	29.60 (28.50–30.80)	30.10 (28.93–31.00)	0.004
	< 27	77 (7.7)	0.394	15 (9.2)	38 (7.1)	24 (7.9)	0.684
MCHC, gL	316–354	337.0 (331.0–344.0)	0.234	337.0 (329.3–342.8)	337.0 (331.0–344.0)	337.0 (332.0–344.8)	0.575
	< 316	31 (3.1)	0.352	7 (4.3)	15 (2.8)	9 (3.0)	0.903
RDW, %	11–16	12.30 (11.90–13.00)	0.658	12.30 (11.90–13.00)	12.30 (11.90–13.00)	12.20 (11.80–13.00)	0.414
	>16	24 (2.4)	0.778	5 (3.1)	13 (2.4)	6 (2.0)	0.664
Platelet, × 10^9^/L	125–350	212.0 (176.0–254.0)	0.018	196.0 (167.0–244.5)	212.0 (176.0–256.0)	219.0 (182.3–256.0)	0.411
	< 125	38 (3.8)	0.014	13 (7.9)	18 (3.4)	7 (2.3)	0.380
MPV, fL	7.4–12.5	9.60 (8.50–10.50)	0.021	9.80 (8.90–10.60)	9.50 (8.30–10.50)	9.40 (8.50–10.40)	0.889
	>12.5	29 (2.9)	0.795	5 (3.1)	15 (2.8)	9 (3.0)	0.903
PDW, %	9–17	15.80 (11.90–16.40)	0.004	14.10 (11.40–16.20)	15.90 (12.20–16.40)	16.00 (12.00–16.60)	0.427
	>17	77 (7.7)	0.225	15 (9.2)	34 (6.4)	28 (9.2)	0.133
**Coagulation factors**							
Prothrombin time, s	9–13	11.50 (10.80–12.50)	0.053	11.35 (10.60–12.38)	11.50 (10.90–12.50)	11.40 (10.70–12.50)	0.139
	>13	136 (13.6)	0.432	20 (12.2)	78 (14.6)	38 (12.5)	0.390
INR	0.8–1.2	0.98 (0.92–1.06)	0.018	0.97 (0.90–1.03)	0.98 (0.92–1.07)	0.98 (0.92–1.05)	0.095
	>1.2	49 (4.9)	0.088	5 (3.1)	36 (6.8)	8 (2.6)	0.010
APTT, s	20–40	30.80 (27.20–34.00)	0.008	29.90 (26.53–33.58)	31.50 (28.00–34.90)	30.30 (26.20–33.30)	< 0.0001
	>40	33 (3.3)	0.508	7 (4.3)	17 (3.2)	9 (3.0)	0.854
Thrombin time, s	14–21	16.00 (14.30–19.00)	< 0.0001	18.35 (14.90–19.50)	15.60 (14.30–18.90)	15.50 (14.00–18.90)	0.208
	>21	13 (1.3)	0.025	6 (3.7)	5 (0.9)	2 (0.7)	1.000
Fibrinogen, g/L	2–4	2.75 (2.30–3.24)	0.107	2.65 (2.15–3.16)	2.75 (2.27–3.19)	2.81 (2.35–3.37)	0.009
	>4	37 (3.7)	0.427	7 (4.3)	16 (3.0)	14 (4.6)	0.230
D-dimer, μg/L	< 550	220.0 (150.0–350.0)	0.001	260.0 (184.2–439.6)	220.0 (152.6–340.0)	210.0 (142.4–337.5)	0.117
	>550	108 (10.8)	0.001	29 (17.7)	47 (8.8)	32 (10.5)	0.416
**Metabolic panel**							
CRP, mg/L	0–10	5.00 (1.40–9.30)	0.188	3.50 (1.60–8.18)	5.00 (1.18–9.46)	5.00 (1.60–9.57)	0.497
	>10	226 (22.6)	0.281	31 (18.9)	122 (22.9)	73 (24.0)	0.712
Procalcitonin, ng/mL	< 0.5	0.15 (0.10–0.30)	0.751	0.16 (0.10–0.25)	0.16 (0.10–0.33)	0.14 (0.10–0.25)	0.189
	>0.5	157 (15.7)	0.967	29 (17.7)	95 (17.8)	33 (10.9)	0.007
Total bilirubin, μmol/L	3–22	8.80 (6.39–12.70)	0.036	8.00 (5.70–11.28)	8.70 (6.21–12.23)	9.62 (7.00–13.90)	0.002
	>22	39 (3.9)	0.580	6 (3.7)	15 (2.8)	18 (5.9)	0.026
Direct bilirubin, μmol/L	0–5	2.29 (1.31–3.36)	0.075	2.44 (1.20–3.80)	2.11 (1.31–3.20)	2.50 (1.41–3.40)	0.011
	>5	51 (5.1)	0.062	11 (6.7)	18 (3.4)	22 (7.2)	0.012
Indirect bilirubin, μmol/L	0–19	6.60 (4.13–9.91)	0.003	5.50 (3.05–8.87)	6.50 (4.21–9.75)	7.38 (4.50–11.27)	0.013
	>19	41 (4.1)	0.674	6 (3.7)	16 (3.0)	19 (6.3)	0.024
ALT, U/L	9–50	26.00 (19.00–36.00)	0.032	28.00 (21.00–37.75)	25.00 (18.00–35.50)	26.00 (20.00–37.00)	0.149
	>50	125 (12.5)	0.735	21 (12.8)	63 (11.8)	41 (13.5)	0.482
AST, U/L	15–40	24.00 (20.00–30.00)	0.024	27.00 (21.00–34.50)	25.00 (21.00–30.00)	23.00 (20.00–27.00)	0.004
	>40	90 (9.0)	0.007	25 (15.2)	43 (8.1)	22 (7.2)	0.666
AST/ALT	0.6–1.5	0.92 (0.71–1.22)	0.520	0.93 (0.73–1.20)	0.96 (0.74–1.29)	0.88 (0.69–1.10)	< 0.001
	< 0.6		0.165	12 (7.3)	59 (11.1)	42 (13.8)	0.241
	>1.5		0.227	24 (14.6)	100 (18.8)	23 (7.6)	< 0.0001
ALP, U/L	38–126	75.00 (61.00–100.00)	0.762	83.00 (61.00–114.50)	79.00 (63.00–114.50)	72.00 (60.00–85.00)	< 0.0001
	>126	168 (16.8)	0.620	36 (22.0)	127 (23.8)	5 (1.6)	< 0.0001
GGT, U/L	12–43	18.00 (13.00–29.00)	0.320	18.50 (13.00–31.00)	18.00 (13.00–27.00)	19.00 (14.00–29.00)	0.007
	>43	120 (12.0)	0.110	24 (14.6)	54 (10.1)	42 (13.8)	0.108
Total protein, g/L	63–82	73.20 (69.30–77.85)	0.411	72.50 (68.53–77.58)	73.00 (69.05–77.70)	74.10 (70.13–78.58)	0.102
	< 63	45 (4.5)	0.038	13 (7.9)	21 (3.9)	11 (3.6)	0.816
Albumin, g/L	35–50	45.80 (43.35–48.10)	< 0.001	44.80 (42.40–47.18)	46.00 (43.60–48.15)	46.00 (43.40–48.48)	0.851
	< 35	4 (0.4)	1.000	0 (0)	2 (0.4)	2 (0.7)	0.624
Globulin, g/L	20–30	27.40 (24.40–30.80)	0.412	27.50 (24.50–30.90)	27.10 (24.15–30.60)	27.90 (24.63–31.20)	0.078
	< 20	54 (5.4)	0.532	8 (4.9)	33 (6.2)	13 (4.3)	0.242
BUN, mmol/L	2.5–6.1	4.44 (3.73–5.40)	0.023	4.51 (3.86–5.76)	4.43 (3.69–5.26)	4.43 (3.71–5.48)	0.312
	>6.1	128 (12.8)	0.005	30 (18.3)	54 (10.1)	44 (14.5)	0.060
Creatinine, μmol/L	46–92	57.00 (45.80–70.80)	0.125	58.50 (45.28–73.40)	54.90 (43.55–68.10)	61.95 (50.53–73.00)	< 0.0001
	>92	40 (4.0)	0.249	8 (4.9)	16 (3.0)	16 (5.3)	0.101
LDH, U/L	120–246	196.0 (173.0–230.0)	0.021	207.0 (178.0–247.8)	195.0 (174.0–231.0)	192.5 (171.0–221.5)	0.156
	>246	182 (18.2)	0.021	42 (25.6)	93 (17.5)	47 (15.5)	0.459
CPK, U/L	30–135	78.00 (53.50–115.00)	0.840	79.50 (51.00–120.80)	77.00 (53.00–114.50)	80.00 (55.25–115.80)	0.570
	>135	161 (16.8)	0.821	28 (17.1)	87 (16.3)	46 (15.1)	0.650
Glucose, mmol/L	3.89–6.11	5.86 (5.27–6.60)	0.006	6.10 (5.30–6.80)	5.73 (5.20–6.50)	5.90 (5.40–6.64)	0.013
	>6.11	381 (38.1)	0.002	77 (47.0)	179 (33.6)	125 (41.1)	0.029
Cholesterol, mmol/L	2.89–5.2	4.54 (3.94–5.21)	0.919	4.45 (3.92–5.08)	4.45 (3.83–5.15)	4.70 (4.16–5.45)	< 0.0001
	>5.2	253 (25.3)	0.348	33 (20.1)	126 (23.6)	94 (30.9)	0.021
Triglyceride, mmol/L	0.7–1.7	1.01 (0.68–1.48)	0.042	1.07 (0.71–1.60)	0.95 (0.66–1.43)	1.07 (0.76–1.53)	0.007
	>1.7	191 (19.1)	0.068	37 (22.6)	87 (16.3)	67 (22.0)	0.040
Potassium, mmol/L	3.5–5.3	3.96 (3.72–4.18)	0.146	3.90 (3.69–4.19)	3.99 (3.73–4.19)	3.91 (3.72–4.15)	0.061
	< 3.5	92 (9.2)	0.012	22 (13.4)	38 (7.1)	32 (10.5)	0.088
Sodium, mmol/L	137–145	137.9 (135.4–140.6)	0.195	138.3 (135.4–140.6)	137.7 (135.4–140.3)	138.3 (135.5–141.0)	0.053
	< 137	399 (39.9)	0.172	60 (36.6)	227 (42.6)	112 (36.8)	0.103
Total calcium, mmol/L	2.1–2.55	2.34 (2.27–2.42)	0.004	2.32 (2.24–2.40)	2.35 (2.27–2.42)	2.34 (2.27–2.42)	0.422
	< 2.1	20 (2.0)	0.012	8 (4.9)	8 (1.5)	4 (1.3)	1.000

For each parameter, the patient number (N) and proportion (%) with abnormal values were calculated and indicated as N (%). Comparisons were performed between patients none/partially vaccinated and patients fully vaccinated (exhibited by *p*^1^ values), between patients fully vaccinated and patients who were three doses vaccinated (exhibited by *p*^2^ values).

WBCs, white blood cells; RBCs, red blood cells; HCT, hematocrit; MCV, mean corpuscular volume; MCH, mean corpuscular hemoglobin; MCHC, mean corpuscular hemoglobin concentration; RDW, red blood cell distribution width; MPV, mean platelet volume; PDW, platelet distribution width; INR, international normalized ratio; APTT, activated partial thromboplastin time; CRP, c-reactive protein; ALT, alanine aminotransferase; AST, aspartate aminotransferase; ALP, alkaline phosphatase; GGT, γ-glutamyl transferase; BUN, blood urea nitrogen; LDH, lactic dehydrogenase; CPK, creatine phosphokinase.

Simultaneously, most biochemical/metabolic indicators in the omicron COVID-19 patients revealed a mild virulent impact, where less than 10% patients showed aberrant values. Nevertheless, it was marked that the portions of patients with aberrant values of several hematological indices were still considerable, especially those including c-reactive proteins (CRPs), procalcitonin, alanine aminotransferase (ALT), alkaline phosphatase (ALP), γ-glutamyl transferase (GGT), blood urea nitrogen (BUN), lactic dehydrogenase (LDH), creatine phosphokinase (CPK), glucose, cholesterol, triglyceride, and sodium. Those results indicated that the infection of the omicron variant still caused noticeable injuries on major organs, such as liver and heart.

Compared to patients who were none/partially vaccinated, patients fully vaccinated exhibited a significant improvement in their hematological profile, including mitigations in thrombocytopenia, thrombin time prolonging, D-dimer elevation, deranged metabolic biomarkers (such as ALT, AST, BUN, and LDH), and electrolyte imbalances. Moreover, patients with booster vaccination showed undifferentiable hematological patterns from patients with full vaccination, despite of some alleviated characteristics such as AST, ALP, and creatinine.

### Hepatic dysfunction in the COVID-19 patients infected by the omicron variant of SARS-CoV-2

Conventional scoring systems for advanced liver diseases, including APRI, FIB-4, and MELD, were brought here to estimate the hepatic dysfunction in COVID-19 patients using their clinical characteristics upon hospital admission. To assess their prognostic value in COVID-19 severity and mortality, we first employed those scoring systems in the wild-type SARS-CoV-2 infected patients admitted in Wuhan, China, in 2020, where 240 mild cases, 88 severe survivals and 72 severe deaths were included and evaluated for liver dysfunction (Supplementary Table S1). Compared to severe survival cases, mild COVID-19 patients possessed significantly lower APRI, FIB-4, and MELD values, while severe deceased patients exhibited comparable values of APRI and MELD scores (although FIB-4 score was higher). At the same time, we also compared the wild-type SARS-CoV-2 infected patients who had raised ALT or AST more than three times the upper limit unit of normal (ULN), or had elevated ALP, GGT, or total bilirubin (TBIL) greater than two times the ULN, between groups of different disease severities. Results point out that the scoring system predicting liver dysfunction is of prognostic value for COVID-19 severity but not mortality in the wild-type SARS-CoV-2 infection.

Next, we applied those scores to compare the liver dysfunctions in all mild cases between the wild-type, delta variant and omicron variant infections. Results are shown in Table 3. Except for the negative median MELD values in omicron infected patients that is invalid, APRI and FIB-4 scores demonstrated the alleviated liver damages in omicron infection compared to the delta variant or the wild-type infection. Besides, patients with raised ALT/AST>3 × ULN or ALP/GGT/TBIL>2 × ULN took up marginal proportions in each infection cohort, so the comparison based on such evaluation could be least meaningful.

**Table 3:** Scoring systems that are conventionally evaluated for advanced liver diseases, including AST to platelet ratio index (APRI), Fibrosis-4 (FIB-4), and Model for End-stage Liver Disease (MELD), or raised AST/ALT values greater than three times of the upper limit of the normal (ULN), or elevated ALP/GGT/total bilirubin (TBIL) values greater than two times of the ULN, were compared between patients with the omicron variant infection and patients with the delta variant infection (exhibited by *p*^OD^ values), or between patients with the omicron variant infection and patients with the wild-type SARS-CoV-2 infection (exhibited by *p*^OW^ values), or between patients with the delta variant infection and patients with the wild-type SARS-CoV-2 infection (exhibited by *p*^DW^ values).

**Scoring**	***p* ^DW^**	***p* ^OD^**	**Delta mild (*n* = 334)**	**Omicron mild (*n* = 1001)**	**Wild type mild (*n* = 240)**	***p* ^OW^**
APRI	0.010	0.0001	0.325 (0.225–0.569)	0.290 (0.227–0.395)	0.298 (0.185–0.525)	0.841
FIB−4	< 0.001	< 0.0001	1.534 (0.849–2.762)	0.783 (0.478–1.237)	1.155 (0.711–2.019)	< 0.0001
MELD	< 0.0001	< 0.0001	1.927 (−0.411–4.999)	−0.360 (−3.302–2.546)	3.754 (1.479–6.557)	< 0.0001
	*p* ^DW^	*p* ^OD^	**Delta mild (** ***N*** **, %)**	**Omicron mild (** ***N*** **, %)**	**Wild type mild (** ***N*** **, %)**	*p* ^OW^
ALT>3 × ULN	1.000	1.000	2 (0.6)	7 (0.7)	2 (0.8)	0.688
AST>3 × ULN	0.289	0.376	3 (0.9)	4 (0.4)	5 (2.1)	0.017
ALP>2 × ULN	0.513	< 0.0001	2 (0.6)	65 (6.5)	0 (0)	< 0.0001
GGT>2 × ULN	0.094	0.187	10 (3.0)	18 (1.8)	14 (5.8)	< 0.001
TBIL>2 × ULN	0.005	0.156	2 (0.6)	1 (0.1)	10 (4.2)	< 0.0001

## Discussion

Previous research established that the pre-existing liver diseases (e.g., hepatitis, cirrhosis, non-alcoholic fatty liver diseases) constitute risk factors for COVID-19 susceptibility, severity, and mortality, due to the exacerbated inflammatory response and worsened immune dysfunction (26, 27). In contrast, the large-size cohort studies on clinical characteristics of COVID-19 indicated that despite of 0.6–2.1% patients with liver disease comorbidity, 21.3–58.4% patients upon hospital admission presented abnormal liver biochemistry (e.g., ALT, AST) (28–30). We here investigated the putative liver injury caused by infections of SARS-CoV-2 or its evolving variants after exclusion of the COVID-19 patients with known comorbidity of chronic liver and liver-related diseases. Our results indicated a changing severity of liver damage in COVID-19 patients when infected by the wild type, the delta or omicron variant.

COVID-19 associated liver insults have been postulated via either direct or indirect viral hit. Both gene and protein expressions of angiotensin-converting enzyme 2 (ACE2) as the known SARS-CoV-2 receptor for host entry revealed similarly moderate levels in the liver and lung, much lower than that in the gastrointestinal tract (31, 32). Inside the liver tissue, cholangiocytes exhibited the highest expression of viral receptors and facilitators, followed by hepatocytes and sinusoidal endothelial cells (33). Those facts infer a molecular tropism of SARS-CoV-2 to directly infect the liver, further evidenced by visualization of viral particles in the hepatocytes (34). Post-mortem liver wedge biopsy or autopsy reports on COVID-19 death confirmed positive detection of SARS-CoV-2 RNA in nearly 70% of liver specimens, illustrating a direct infection resembling other hepatotropic viruses (e.g., hepatitis C virus) where interferon response was upregulated and JAK/STAT signaling was activated (35, 36). In accordance with those findings, JAK inhibitors reduced liver infectivity of SARS-CoV-2, so lowering the inflammation to ameliorate the COVID-19 progression (37). Nevertheless, hepatic locations of SARS-CoV-2 spike proteins and ACE2 receptors were spatially mismatched, pointing out that viral uptake in the organs does not solely depend on ACE2 receptors (36). Alternatively, SARS-CoV-2 can infect the immune cells and migrate into the liver through the portal veinous system after extended viral shedding in the gut (38).

In parallel to a direct liver impact by SARS-CoV-2 infection, indirect hepatic injury secondary to systemic inflammation and vascular thrombosis occurs and may even be the primary route leading to severe organ failure (39). The large-scale and multicentric COVID-19 autopsy reports reach consensus that the most pathological lesions were concentrated in lungs, including diffuse alveolar damage, alveolar–capillary barrier damage, and increased vascular permeability, followed by multiorgan failures that were usually featured by blood coagulopathy and microthrombi formation in extrapulmonary tissues including livers (40, 41). Thereby, the liver injury in COVID-19 patients is acute but transient and mild, and commonly with minimal inflammation, albeit the fatal liver damages have also been sparsely reported in the previously heathy COVID-19 patients (42, 43). Insofar, health outcomes following 6, 12, and 24 months after disease onset have not specified the expansive liver-related consequence in long COVID cases (44); however, increased liver fibrogenesis was notified in patients 3–6 months post COVID (45).

With a genomic length of 30,000 nucleotides, SARS-CoV-2 owns a mutation rate at a magnitude of 10^−3^ per nucleotide per year (46), comparable to that of influenza A/B virus (47), or SARS-CoV (48), Middle East Respiratory Syndrome Coronavirus (MERS-CoV) (49), or seasonal human CoVs (50). C-to-U conversion was found prevalent in the mutations of SARS-CoV-2 (51), implying its RNA editing by deaminases like APOBEC family enzymes in the viral host, embracing fitness advantages (52, 53). Currently, the dominant omicron variant carries mutations from previous VOCs, including L452R, N501Y, and D614G, while unique variations (e.g., Q498R and N679K) contribute to substantially elevated transmission and immune evasion, possibly due to much increased host binding affinity and decreased antibody neutralizing ability (54, 55).

Paradoxically, infections by the omicron variant led to a milder intrinsic severity when compared to those by the earlier variants (56–60). This could be explained by the less involvement in the lower respiratory tract of the omicron patients (61). Although omicron develops a higher affinity for human ACE2, its cell entry route follows an endocytic pathway and is independent of membrane-bound protease priming, which is distinctive from other SARS-CoV-2 variants (62, 63). This adaptation not only renders the omicron variant a broader spectrum of cellular tropism to infect ACE2^+^ cells that are more abundant in human bronchi than lungs, but also attenuates its viral replication, leading to mitigated pro-inflammatory responses and diminished lung pathology (64, 65). Those findings stand in line with our results reported here, where more than half proportion of patients went through asymptomatic manifestations and infection did not induce severe pathological changes in the hematological profiles of most patients.

This altered pathogenesis may imply a shifted disease pattern and clinical manifestation in the omicron infection. A lately report indicated that compared to the wild type or the delta variant, the omicron variant exhibited much less capacity of viral replications in intestinal organoids, producing lower levels of pro-inflammatory cytokines (66). This explains much reduced occurrence of gastrointestinal symptoms in the omicron-infected patients who had very low frequency of diarrhea, vomiting or abdominal pain. Similarly, our results here indicated a mitigated liver injury induced by the omicron variant of SARS-CoV-2, when compared to that by the wild type or the delta variant, evaluated by the liver fibrosis scores or key hepatic biomarkers. This result disagrees with others where the omicron liver injury appeared comparable to the delta variant or the wild type, based on the proteomics analysis of patients' sera or the liver function tests in the cohort size of tens (67, 68). However, a recent spreading of severe hepatitis with unknown etiology in children has been identified in association with high population infection of omicron variant (69), and proposed as SARS-CoV-2 triggered immune activation superimposed by adenovirus infection (70). Therefore, the long-term impact of SARS-CoV-2 and its evolving variants on the liver deserves continued and heightened attention.

Our study had several limitations. Firstly, we are in no position to conduct either biopsy or autopsy studies to glean the direct evidence of liver injury, caused by the SARS-CoV-2 omicron variant. It would otherwise greatly enhance our understanding toward the liver tropism and impairment with viral insults. Secondly, due to the mild infection by the omicron variant, there was no severe or deceased patient included in our study, so we had no access to analyze the possible predictors or risk factors for severity or mortality of omicron COVID-19 infection. Thirdly, our study contained patients with a median age of 36.0 (IQR: 26.0–50.0), so we could not elucidate much of viremia in the aged population (> 60 years old). Fourthly, this study lacked a continuous monitoring of COVID-19 patients during hospitalization and in particular, post hospital discharge. A long-term sequalae following the omicron infection to justify its pathogenic feature and consequence would be necessitated.

## Conclusions

In closing, we investigated the clinical characteristics of 1,001 COVID-19 patients infected by the omicron variant of SARS-CoV-2 with no known liver disease comorbidity, finding the reduced severity overall and especially on the livers. Albeit the high mutation in the omicron variant may effectuate its evasion from neutralizing antibodies, the innate and acquired immunity of patients could defense against the viral attack of the omicron variant, attested by a majority of patients being asymptomatic. Simultaneously, the infection route and intrinsic virulence of the omicron variant greatly alter, attenuating its detrimental effect on extrapulmonary organs such as livers.

## Data availability statement

The raw data supporting the conclusions of this article will be made available by the authors, without undue reservation.

## Ethics statement

The studies involving human participants were reviewed and approved by the Fifth People's Hospital of Suzhou (TFPHS, the Affiliated Infectious Diseases Hospital of Soochow University) and the Fourth People's Hospital of Lianyungang (TFPHL), China. Written informed consent from the participants' legal guardian/next of kin was not required to participate in this study in accordance with the national legislation and the institutional requirements.

## Author contributions

ZT conceived the idea and designed the study. JZ, DZ, JH, QG, and ZT contributed to the data processing and table preparation. XH and ZT contributed to the statistical analysis. JZ, DZ, and JH contributed equally to this work. All authors contributed to the manuscript writing and approved the manuscript submission.

## Conflict of interest

The authors declare that the research was conducted in the absence of any commercial or financial relationships that could be construed as a potential conflict of interest.

## Publisher's note

All claims expressed in this article are solely those of the authors and do not necessarily represent those of their affiliated organizations, or those of the publisher, the editors and the reviewers. Any product that may be evaluated in this article, or claim that may be made by its manufacturer, is not guaranteed or endorsed by the publisher.

## Abbreviations

ALP: alkaline phosphatase
ALT: alanine aminotransferase
AST: aspartate aminotransferase
APRI: AST to platelet ratio index
APTT: activated partial thromboplastin time
BUN: blood urea nitrogen
COVID-19: corona virus disease 2019
CPK: creatine phosphokinase
CRP: c-reactive protein
FIB-4: Fibrosis-4
GGT: γ-glutamyl transferase
HCT: hematocrit
INR: international normalized ratio
LDH: lactic dehydrogenase
MCH: mean corpuscular hemoglobin
MCHC: mean corpuscular hemoglobin concentration
MCV: mean corpuscular volume
MELD: model for end-stage liver disease
MPV: mean platelet volume
PDW: platelet distribution width
RBCs: red blood cells
RDW: red blood cell distribution width
SARS-CoV-2: severe acute respiratory syndrome coronavirus 2
TBIL: total bilirubin
ULN: upper limit of the normal
WBCs: white blood cells.
