# Supplementary material for: Hepatic dysfunctions in COVID-19 patients infected by the omicron variant of SARS-CoV-2
Source: Front Public Health. 2022 Nov 18;10:1049006. doi: 10.3389/fpubh.2022.1049006 (PMC9716022; doi:10.3389/fpubh.2022.1049006)

**Hepatic dysfunctions in COVID-19 patients infected by the omicron variant of SARS-CoV-2**

Jianguo Zhang^1,†^, Daguo Zhao^2,†^, Jianhui Hu^3,†^, Xing Huang^4^, Qingqing Gu^5^, Zhimin Tao^1, 6, *^

^1^Department of Emergency Medicine, The Affiliated Hospital of Jiangsu University, Zhenjiang, Jiangsu 212001, China

^2^Department of Critical Care Medicine, The First Affiliated Hospital of Soochow University, Jiangsu 215006, China

^3^Department of Laboratory Medicine, Zhenjiang Hospital Affiliated to Nanjing University of Chinese Medicine, Zhenjiang Hospital of Traditional Chinese Medicine, Jiangsu 212003, China

^4^Center for Evidence-Based and Translational Medicine, Zhongnan Hospital of Wuhan University, Hubei 430071, China

^5^Department of Infectious Diseases, The Affiliated Hospital of Kangda College of Nanjing Medical University, The Fourth People’s Hospital of Lianyungang, Jiangsu 222023, China

^6^Jiangsu Province Key Laboratory of Medical Science and Laboratory Medicine, School of Medicine, Jiangsu University, Zhenjiang, Jiangsu 212013, China

^†A^uthors contributed equally to this work.

*Correspondences should be addressed to:

Zhimin Tao: [jsutao@ujs.edu.cn](mailto:jsutao@ujs.edu.cn)

**Table S1.** Scoring systems that are conventionally evaluated for advanced liver diseases, including AST to platelet ratio index (APRI), Fibrosis-4 (FIB-4), and Model for End-stage Liver Disease (MELD), or raised AST/ALT values greater than three times the upper limit of the normal (ULN), or elevated ALP/GGT/total bilirubin (TBIL) values greater than two times of the ULN, were compared between mild patients and severe patients survived in ICU (exhibited by *p*^12^ values), or between severe patients survived and deceased in ICU (exhibited by *p*^23^ values), in the wild-type SARS-CoV-2 infection. ALT, alanine aminotransferase; AST, aspartate aminotransferase; ALP, alkaline phosphatase; GGT, γ-glutamyl transferase; TBIL, total bilirubin. The scoring systems applied in our study were calculated as below:

APRI$=\frac{{AST (U/L)}/{AST ULN(U/L)}}{Platelet count ({10}^{9}/L)}\times100$ FIB-4 $=\frac{age \left( years \right)\times AST(U/L)}{Platelet count ({10}^{9}/L)\times\sqrt{ALT(U/L)}}$

MELD = 3.78×ln[TBIL(mg/dL)]+11.2×ln(INR)+9.57×ln[creatinine(mg/dL)]+6.43


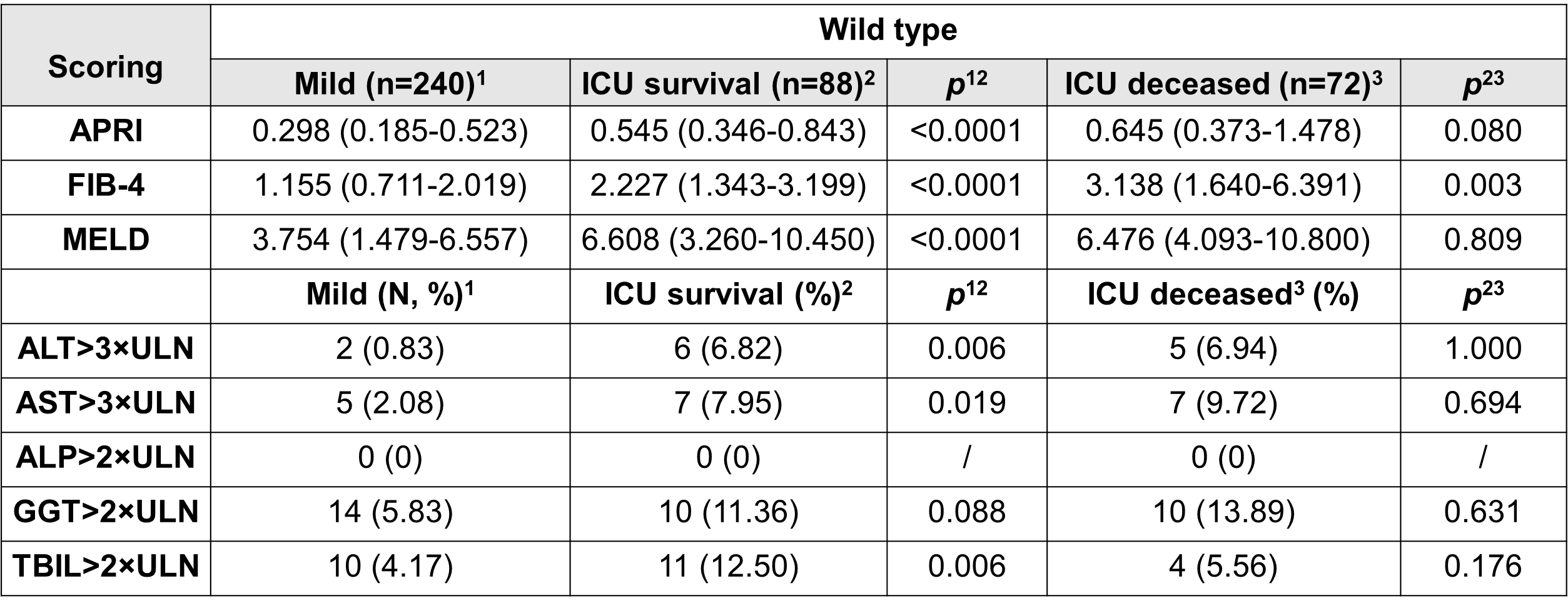

Supplement: Supplementary file 1 [file Table_1.docx]
